# Shark Attacks in Dakar and the Cap Vert Peninsula, Senegal: Low Incidence despite High Occurrence of Potentially Dangerous Species

**DOI:** 10.1371/journal.pone.0001495

**Published:** 2008-01-30

**Authors:** Sébastien Trape

**Affiliations:** 1 Institut de Recherche pour le Développement (IRD), Unité de Recherche 070, Dakar, Sénégal; 2 Laboratoire ECOLAG, Unité Mixte de Recherche 5119, Université Montpellier, Montpellier, France; University of St. Andrews, United Kingdom

## Abstract

**Background:**

The International Shark Attack File mentions only four unprovoked shark attacks on the coast of West Africa during the period 1828–2004, an area where high concentrations of sharks and 17 species potentially dangerous to man have been observed. To investigate if the frequency of shark attacks could be really low and not just under-reported and whether there are potentially sharks that might attack in the area, a study was carried out in Dakar and the Cap Vert peninsula, Senegal.

**Methodology/Principal Findings:**

Personnel of health facilities, administrative services, traditional authorities and groups of fishermen from the region of Dakar were interviewed about the occurrence of shark attacks, and visual censuses were conducted along the coastline to investigate shark communities associated with the coasts of Dakar and the Cap Vert peninsula. Six attacks were documented for the period 1947–2005, including two fatal ones attributed to the tiger shark *Galeocerdo cuvieri*. All attacks concerned fishermen and only one occurred after 1970. Sharks were observed year round along the coastline in waters 3–15 m depth. Two species potentially dangerous for man, the nurse shark *Ginglymostoma cirratum* and the blacktip shark *Carcharhinus limbatus*, represented together 94% of 1,071 sharks enumerated during 1,459 hours of observations. Threatening behaviour from sharks was noted in 12 encounters (1.1%), including 8 encounters with *C. limbatus*, one with *Galeocerdo cuvieri* and 3 with unidentified sharks.

**Conclusions/Significance:**

These findings suggest that the frequency of shark attacks on the coast of West Africa is underestimated. However, they also indicate that the risk is very low despite the abundance of sharks. In Dakar area, most encounters along the coastline with potentially dangerous species do not result in an attack. Compared to other causes of water related deaths, the incidence of shark attack appears negligible, at least one thousand fold lower.

## Introduction

On the coast of West Africa, sharks are well represented, both by the number and species [1], [2], [3], [4], [5]. Although there are a number of species potentially dangerous to man, little data is available concerning shark attacks in this part of the world. The International Shark Attack File (ISAF), the most complete database in this field, mentions four unprovoked shark attacks on the coast of West Africa for the period 1828–2006: two were fatal, in Cape Verde Islands (2001) and in Liberia (1950), and two non fatal, in Senegal and Sierra Leone [6], [7]. By comparison, ISAF reports 212 attacks on the coast of South Africa for the same period, and 365, 94 and 66 attacks for the period 1990–2006 in Florida, Australia and Brazil, respectively [7].

Some authors have questioned the value of these statistics, in particular those concerning the Senegalese coast [8], where high concentrations of sharks have been observed near beaches frequented by both the local population and by numerous tourists. In the literature, only a single case of fatal attack, occurring at Thiaroye, near Dakar, has been documented [9], although seventeen species of sharks well known as potentially dangerous for man constitute the habitual hosts of the Senegalese coast, including the tiger shark *Galeocerdo cuvieri*, the shortfin mako *Isurus oxyrhynchus* and several species of requiem sharks of the genus *Carcharhinus* [2], [3], [10].

To my knowledge, no epidemiological study of shark attacks has been conducted in West Africa. To investigate if the frequency of shark attacks is actually very low and not just under-reported and whether there are potentially sharks that might attack along the coastline in the area, a study was carried out in Senegal's most populated area, the Cap Vert peninsula, where the capital city Dakar is located.

## Materials and Methods

### Study area

**Figure 1:**
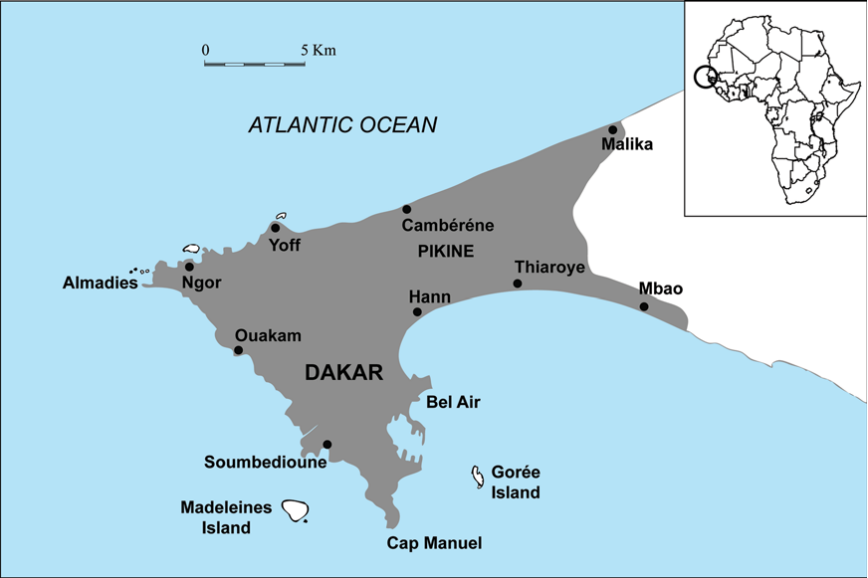
Map of Cap Vert peninsula, Senegal.

The Cap Vert peninsula is located at the westernmost point of Africa (Figure 1). Its littoral presents rocky areas of volcanic origin, distributed in the western part of the peninsula, alternating with sand beaches, where fishing villages of Soumbédioune, Ouakam, Ngor, Yoff, Cambérène, Hann, Thiaroye and Mbao are located. These old villages, where most inhabitants belong to the Lebou ethnic group, have been progressively incorporated into the city of Dakar, the capital of Senegal, and its suburb Pikine. The population of the peninsula in 2005 was estimated at 2,452,000 inhabitants, i.e. approximately one quarter of the population of Senegal [11]. Hydroclimate is characterized by two alternating hydrologic seasons, a season of cold water from January to April (average surface temperatures : 16°C to 18°C), and a season of hot water from July to October (average surface temperatures: 26°C to 28°C) [12].

### Epidemiological investigations

To investigate the frequency and the circumstances of shark attacks, a series of surveys were carried out between November 2005 and August 2006 among health facilities, administrative services, traditional authorities and groups of fishermen from the region of Dakar.

Surveys among all the major health facilities were carried out: the seven public hospitals and health centers of Dakar and its suburbs (Hôpital Principal, Hôpital le Dantec, Centre Hospitalier de Grand Yoff, Centre Hospitalo-Universitaire de Fann, Centre Hospitalier Abass Ndao, Centre de Santé du Roi Baudoin, Centre de Santé Dominique), the four main private health establishments of Dakar (Clinique Pasteur, Clinique des Mamelles, Clinique la Madeleine, Clinique Casahous) and seven dispensaries located near the beaches or fishermen villages of the Cap Vert peninsula (Ngor, Ouakam, Yoff A, Yoff B, Cambérène, Hann and Thiaroye). A letter presenting the objectives of the study was sent to the director of each establishment (except in the case of the dispensaries, where the objectives of the study were directly presented to the head nurse) and the medical personnel that were most likely to have been in contact with victims of a shark attack were systematically interviewed. Information collected on each attack were reported on questionnaires, indicating if a medical file was available, the approximate date of the attack, the severity of the injury and its evolution, the name, age and address of the victim and/or any other useful information for a further interview of the victim or his family.

Interviews with fishermen and traditional authorities on the occurrence of shark attacks were undertaken in the villages of Soumbédioune, Ouakam, Ngor, Yoff, Cambérène, Hann, Thiaroye and Mbao. The common characteristic of these fishermen villages is that they are located on beaches that are widely used for aquatic recreation by the population of Dakar, from June to November. During the rest of the year, the permanent population of these villages are probably the most exposed to shark attacks, due to various underwater fishing activities involving both men and women, mainly spearfishing, sea urchin and shell fishing. The four main places of the peninsula frequented by surfers all year round are located near two of these villages (Ngor and Ouakam). The fire service, state police force, and statistics department from Dakar were also asked about shark attacks. The fire service is generally called for first aid to the drowned and publishes an annual report on the causes of accidents where it intervened.

The study was initially designed to investigate shark attacks that took place over a period of 20 years, from January 1986 to December 2005. However, it rapidly became apparent that most cases mentioned by the personnel of health establishments or by other sources occurred before 1986. Even for these old cases it was still relatively easy to trace the victim, or his family, because of the high emotional impact in the community of such accidents. Thus, it was decided to extend the study to all presumed cases of shark attacks that were mentioned, and to systematically visit at home the victims or their families in order to confirm the responsibility of a shark, and to obtain additional information on the circumstances of the attack and the nature of injuries.

The study protocol was approved by the head or representative of all health establishments or institutions involved in the project. Written informed consent was obtained for the questionnaire from each victim or witness of shark attack. Each individual represented saw a copy of this paper and agree to it being published with their details included.

### Shark Occurrence and Behaviour

The distribution and abundance of sharks and their behaviour on the coasts of the Cap Vert peninsula when encountering humans were investigated by two different surveys:

- a prospective survey, performed in November–December 2005 and in July–August 2006 by snorkelling along transects parallel to the coast line at the Almadies, Ngor, Mbao, Madeleine Island and Gorée Island where sharks were enumerated by visual census in waters 3–15 m depth. 57 censuses were made, totaling 169 hours of observations (northern coast: 50 censuses, 145 hours; southern coast: 7 censuses, 24 hours). Each census lasted approximately 3 hours and the distance covered was between 1.5 and 2 km. In most cases visibility ranged from 7 to 12 m.

- a retrospective survey, bearing on one thousand encounters with sharks during spearfishing or snorkelling along the coast of the Cap Vert peninsula between October 1986 and April 2005. All observations were performed less than 500 m from the coast line of the peninsula or nearby islands (Madeleines, Gorée, Ngor, Yoff) or reefs (Almadies) in waters 3–15 m deep. They were performed year round, generally on a weekly basis, most of them at the Almadies (91% of the total number of hours of observations on the northern coast) and Gorée Island (73% of the total number of hours of observations on the southern coast). In most cases visibility ranged from 7 to 12 m.

Identification of the species of sharks was generally possible for the two most common species in Cap Vert peninsula waters by using simple morphological or behavioural criteria: the blacktip shark *Carcharhinus limbatus* differs from all other West African Carcharhinidae species by the presence of a well marked dark band on the flanks, from the level of the pectoral fin to the level of the ventral fin; the nurse shark *Ginglymostoma cirratum* is generally observed lying on sandy bottoms. Except for a few number of specimens of small size that were harpooned, sharks belonging to other species were generally not identified to the species, but most of them were Carcharhinidae. The duration of each census, the number of sharks from each species, and behavioural observations were consigned daily during the prospective survey. For the retrospective survey, the total number of hours of observations was estimated from the number of days with snorkelling or spearfishing activities and the average time spent snorkelling or spearfishing on those days.

## Results

### Epidemiological investigations

Fifteen case histories of shark attacks were collected during investigations in health facilities or fishermen villages. Nine cases were excluded from this study: five cases because injuries occurred on a pirogue or either on the beach after the shark was captured, two cases that occurred along the coastline in another region of Senegal (Kayar, 45 km NE of Dakar), and one case which corresponded to an accident with dynamite used for fishing, that was presented as a shark attack for the transport of the victim to hospital.

**Figure 2:**
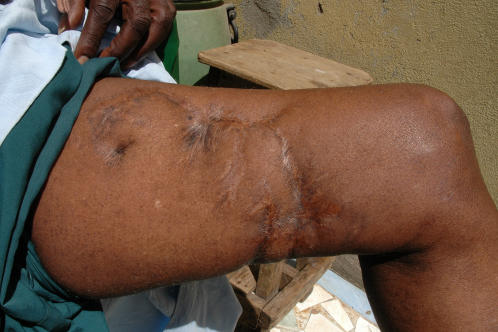
The thigh of a Senegalese fisherman forty years after a shark attack.

Six attacks that occurred along the coastline of the Cap Vert peninsula were documented. Two of them were fatal and all occurred among fishermen between 1947 and 1998. Except for the fatal shark attack previously published by Dejou & Almeida that occurred in 1947 [9], it has been possible to trace and to directly interview all the victims who survived (4 attacks) or to interview a person who assisted the victim in the sea immediately after the shark attack (one fatal attack). The five case histories obtained from these interviews are as follows:

- Patient A. N., a Lebou fisherman, twenty year-old, was bitten at the abdomen by a shark in 1961. The attack occurred at approximately 09:00 h at 300–500 m from the beach of Thiaroye Guedj. The victim was diving in apnoea in 5–7 m of water for fishing molluscs of the genera *Cymbium*. He returned to the surface with a *Cymbium* in his hands when the attack of a large shark (>3 m) occurred. The abdomen of the victim was lacerated and part of the intestines was hanging out when he was hoisted on the pirogue. He died a few hours later at the Thiaroye health center.

- Patient M. G., a Lebou fisherman from Yoff, 23 year-old, was bitten at the ankle during beach seine fishing in August 1962. The accident occurred in front of the beach of Malika, between 13:00 h and 14:00 h, at a few dozen meters from the shore, in one meter of water. The fisherman was moving inside the area encircled by the seine, in order to heighten the net for preventing mullets to escape, when the attack happened. The shark had a beige colour and measured approximately 3.5 m. The victim was taken to Le Dantec hospital and recovered from the wound.

- Patient O. D., a Lebou fisherman from Thiaroye Guedj, about thirty year-old, was successively bitten on the thigh and the hand in March 1964. The attack occurred in front of Bel Air, between 12:00 h and 13:00 h, at approximately 600 m from the shore, in 6–8 m of water. The fisherman was returning to the surface with a *Cymbium* in his hands when the first attack to the thigh occurred. The second attack to the hand occurred a few seconds later when the victim was swimming to the pirogue after surfacing. The shark was estimated at least 3 m long, and was the biggest ever seen by this fisherman. The victim was taken to Le Dantec hospital where he remained hospitalized during two months. Forty year later, the scars are still very apparent (Figure 2). The largest scar, in the shape of half-circle on the thigh, is 18 cm diameter.

- Patient B. D., a Lebou fisherman from Hann, was bitten at the ankle in August 1967, during beach seine fishing, in similar circumstances than those of patient M. G., when he was moving in the water surrounded by the seine. The accident occurred at night, in front of the beach of Hann, at about 04:00 h. The shark was approximately 1.8 m. The victim was taken to Hann dispensary and rapidly recovered.

- Patient A. D., a 25 year-old spare fisherman, was bitten at the right calf in September 1998 by the shark he just harpooned near Yoff island. It was a small shark (approximately 1.5 m). The victim was taken to the Centre Hospitalier Abass Ndao and rapidly recovered.

### Shark Occurrence and Behaviour

#### Prospective study

I counted 48 sharks belonging to four different species in 90 hours of snorkelling at the Almadies and Ngor in November-December 2005. They were 22 blacktip sharks *Carcharhinus limbatus* (45.8%), 24 nurse sharks *Ginglymostoma cirratum* (50.0%) and two specimens belonging to two unidentified species (4.2%). All blacktip sharks were adults about 1.8–2.5 m long, and the nurse sharks measured between 1.3 and 1.8 m. The two unidentified specimens measured between 2 and 2.5 m. Out of 48 sharks encountered, 45 were indifferent to my presence (93.7%), two changed direction in order to approach me then stayed at short distance away (5–7 m) for approximately a minute before to moving off (unidentified shark) or until I felt sufficiently threatened to return to the accompanying boat (*Carcharhinus limbatus*) (4.2%), and another *Carcharhinus limbatus* immediately charged with sudden half-turn only two meters from me (2.1%).

I counted 23 sharks belonging to three or more species in 79 hours of snorkeling at the Almadies, Ngor, Mbao, Madeleine Island and Gorée Island in July–August 2006. They were 13 blacktip sharks *Carcharhinus limbatus* (56.6%), 5 nurse sharks *Ginglymostoma cirratum* (21.7%) and five specimens belonging to unidentified species (21.7%). All blacktip sharks were adults about 1.6–2.0 m long and the nurse sharks measured between 1.5 and 2.2 m. Four out of five unidentified specimens measured between 2.0 and 2.5 m and belonged probably to the genus *Carcharhinus*. Out of 23 sharks encountered, all seemed indifferent to my presence.

#### Retrospective study

Out of one thousand encounters with sharks on the coasts of the Cap Vert peninsula during 1290 hours of snorkelling or spearfishing between 1986 and 2005, 715 (71.5%) concerned the nurse shark *Ginglymostoma cirratum* (0.8–2 m), 226 (22.6%) the blacktip shark *Carcharhinus limbatus* (1.7–2.5 m), 11 (1.1%) the barbeled houndshark *Leptocharias smithii* (<1 m), 2 (0.2%) the milk shark *Rhizoprionodon acutus* (<1 m), one (0.1%) the tiger shark *Galeocerdo cuvieri* (4 m), and 45 (4.5%) unidentified sharks (0.8–3 m). None of these encounters were followed by an attack, but in nine cases (0.9%) a threatening behaviour from the shark was observed: three sharks immediately charged then suddenly turned back at only 2–4 m (one *Galeocerdo cuvieri*, one *Carcharhinus limbatus* and one *Carcharhinus sp*.), and six sharks approached progressively to shorter and shorter distances (2–5 m) before eventually moving off (five *Carcharhinus limbatus* and one specimen not identified). These nine encounters occurred during spearfishing activities, but in only two cases had fish been previously harpooned (two cases of progressive approach by *Carcharhinus limbatus*). Almost all encounters with sharks (99%) took place on the northern coast of the Cap Vert peninsula (Almadies, Ngor, Yoff), although the duration of observations in this area were about 850 hours, i.e. only two-third of the whole duration of observations (average: 1.16 shark per hour). Only eleven encounters with sharks (eight *Ginglymostoma cirratum*, two *Rhizoprionodon acutus* and a small unidentified shark) took place on the southern coast of the Cap Vert peninsula, although an estimated 440 hours of observations were made in Mbao, Bel Air, Gorée island, Cap Manuel, Madeleines island, Soumbedioune or Ouakam (average: 0.025 shark per hour).

## Discussion

Five shark attacks were documented during this study, raising to six the total number of shark attacks reported from the coast of the Cap Vert peninsula. Only one case of shark attack in the region of Dakar was previously reported [9]. The victim, a Lebou fisherman who was diving in apnoea in front of the beach of Thiaroye, was bitten at the thigh in December 1947. He died the same day at Le Dantec hospital. Haemorragic shock was the cause of his death, as in most fatal attacks [13].

The methodology used for the epidemiological surveys was mainly based on the interview of a limited number of persons that was most likely informed of the occurrence of shark attacks. The few number of cases documented and the fact that most of them occurred more than 20 years ago suggest that the incidence of shark attacks in the region of Dakar is low. In most health establishment, all medical personnel in contact with victims of accidents were interviewed, including several nurses working for more than 30 years in the same hospital. Surveys among fishermen and traditional authorities showed that many years after they occurred, the cases reported in this study were still well known from many persons in the community, due to the high emotional impact of shark attacks. No case was known by the Dakar fire service, which has responsibilities for accidents on beaches and transports to hospital more than one hundred drowned persons each year, nor from the other administrative sources that were interviewed.

According to ISAF classification criteria, three attacks, of which two were fatal, fall into the category of unprovoked attacks [7], [14]. It was the two attacks directed against the divers fishing for *Cymbium*, and the attack reported by Dejou & Almeida [9]. These three attacks occurred on the southern coast of the Cap Vert peninsula, where sharks are currently less numerous than on the northern coast. According to the fishermen from Hann and Thiaroye, sharks were formerly very abundant in this area, which was selected in the 1940s for the settlement of the first shark fisheries in Senegal [15]. The present rarity of sharks on the southern coast of the Cap Vert peninsula is probably due to the considerable development of traditional fishing since the 1970s on this coast well sheltered from the dominant winds [16]. Sharks are still numerous on the northern coast of the Cap Vert peninsula, but the frequency of encounters with sharks was higher during the period 1986–1997 (0.98 per hour for all areas, 1.42 per hour for the northern coast) than during the period 1998–2006 (0.53 per hour for all areas, 0.76 per hour for the northern coast) (p<0.0001 by exact test).

Seventeen out of 54 species of sharks recorded on the Senegalese coasts are known to have attacked humans (Table 1). The tiger shark *Galeocerdo cuvieri* is considered to be one of the most dangerous species in this region due to its presence all year round and the large size of most specimens encountered on the Senegalese coasts [17]. Next to the great white shark *Carcharodon carcharias*, which is rare in Senegalese waters [5], [10], *G. cuvieri* is the shark which is responsible of the highest number of attacks in the world [7]. The fatal attack of Thiaroye in 1947 was attributed to this species, and the stomach of a tiger shark captured in Joal, 90 km south of Dakar, contained an human foot which is still preserved at the Gorée island Sea Museum [18]. In the present study, the encounter with a tiger shark in front of the Almadies was immediately followed by a charge. The large size -more than 3 meters- of the sharks responsible of the two unprovoked attacks of Bel Air and Thiaroye suggests that tiger sharks could also be involved. Two other large species, the bull shark *Carcharhinus leucas* and to a lesser extent the dusky shark *Carcharhinus obscurus* are both abundant in Senegalese waters and well known for being occasionally responsible for attacks near the coastline in tropical waters [3], [7], [19], [20]. However, all specimens from these two species which were measured on the Senegalese coasts were less than 2.9 meters long [2]. Furthermore, they seem to be rare along the coast of the Cap Vert peninsula, preferring the sandy coast of southern Senegal and the mouth of the Sine-Saloum, Gambia and Casamance rivers.

**Table 1:** List of sharks known from the Senegalese coasts. Species implicated in attacks around the world are indicated in bold (compiled data from references [2], [3], [4], [7], [19], [20]).

Family CHLAMYDOSELACHIDAE	Family ALOPIIDAE
*Chlamydoselachus anguineus*	*Alopias vulpinus*
	*Alopias superciliosus*
Family HEPTRANCHIDAE	
*Heptranchias perlo*	Family CETORHINIDAE
	*Cetorhinus maximus*
Family HEXANCHIDAE	
*Hexanchus griseus*	Family LAMNIDAE
	***Carcharodon carcharias***
Family ECHINORHINIDAE	***Isurus oxyrinchus***
*Echinorhinus brucus*	*Isurus paucus*
	
Family DALATIIDAE	Family SCYLIORHINIDAE
*Etmopterus pusillus*	*Galeus polli*
*Etmopterus spinax*	*Scyliorhinus canicula*
*Oxynotus centrina*	*Scyliorhinus stellaris*
*Oxynotus paradoxus*	
*Scymnodon obscurus*	Family PSEUDOTRIAKIDAE
*Centroscymnus cryptacanthus*	*Pseudotriakis microdon*
*Centroscymnus coelolepis*	
*Centroscymnus crepidater*	Family LEPTOCHARIIDAE
*Scymnorhinus licha*	*Leptocharias smithi*
*Isistius brasiliensis*	
	Family TRIAKIDAE
Family SQUALIDAE	*Mustelus mustelus*
*Squalus blainvillei*	
*Squalus acanthias*	Family HEMIGALEIDAE
	*Paragaleus pectoralis*
Family CENTROPHORIDAE	
*Centrophorus granulosus*	Family CARCHARHINIDAE
*Centrophorus lusitanicus*	***Galeocerdo cuvieri***
*Centrophorus uyato*	***Prionace glauca***
*Centrophorus squamosus*	***Negaprion brevirostris***
*Deania calceus*	*Rhizoprionodon acutus*
	*Carcharhinus signatus*
Family GINGLYMOSTOMATIDAE	***Carcharhinus falciformis***
***Ginglymostoma cirratum***	***Carcharhinus brevipinna***
	***Carcharhinus limbatus***
Family RHINCODONTIDAE	***Carcharhinus longimanus***
*Rhincodon typus*	***Carcharhinus obscurus***
	*Carcharhinus altimus*
Family ODONTASPIDIDAE	***Carcharhinus plumbeus***
***Carcharias taurus***	***Carcharhinus leucas***
	
Family PSEUDOCARCHARIIDAE	Family SPHYRNIDAE
*Pseudocarcharias kamoharai*	***Sphyrna mokarran***
	***Sphyrna zygaena***
	***Sphyrna lewini***

Two species of sharks are remarkably abundant on the northern coast of the Cap Vert peninsula: the nurse shark *Ginglymostoma cirratum* and the blacktip shark *Carcharhinus limbatus*. The former is generally encountered when lying on sandy bottoms, often partially hidden by rocks. It has a placid temperament and most attacks reported in the literature occurred after it was injured or harassed [7], [19], [20]. The latter is known for occasionally attacking humans, but fatal accidents attributable to this species seem to be rare [7], probably because its relatively modest size, the largest specimens rarely reaching 2.5 m [2], [3]. Eight of the 261 blacktip sharks (3.1%) presented a threatening behaviour, including two sudden charges. However, full attacks from this species are probably very rare since no case was reported by the Senegalese spearfishermen and the surfers who daily frequent the coasts of Ngor and the Almadies where this species is abundant most of the year. The apparent paradox of high abundance of sharks and low rate of attacks along the coast of the Cap Vert peninsula is probably due to the relative rarity of the three most dangerous species: *G. cuvieri*, *C. leucas* and *C. carcharias*, compared to the abundance of the much less dangerous *G. cirratum* and *C. limbatus*.

The population of the Cap Vert peninsula has increased 10 fold since 1955. Major sociological changes have occurred during the last decades, and some of them have already had important implications for the exposure of the population of Dakar to sea hazards. Fishing activities have involved more and more people since the 1970's, but the most important change has occurred in the 1990's when bathing had become popular among teenagers and young adults. From June to November, the beaches of Dakar are now as crowded as any beach of a tourist resort in Northern America or Europe. Fire service of Dakar annual reports indicate that 322 deaths by drowning occurred on the beaches of the Cap Vert peninsula during the period 2001–2006, i.e. an average yearly incidence approximately twice the estimates of the total number of fatalities by shark attacks worldwide each year [3]. Since only two fatalities by shark attack were documented for the Cap Vert peninsula over a sixty year period, this suggests that shark attacks may represent in this area less than one per thousand of the number of water related deaths.

In most countries of tropical Africa, there is no system of registration for the causes of morbidity and mortality. Like other causes of accident, the incidence of shark attacks is poorly known, and only four cases are mentioned in the ISAF database for the whole West Africa for the period 1828–2004. Although not exhaustive, our study shows that the frequency of shark attacks on the Senegalese coasts has been underestimated. However it also shows that the frequency of unprovoked attacks is low despite the abundance of sharks. Compared to other causes of water related deaths in Dakar region, shark attack appears negligible.
